# An Assessment of Vitreous Degeneration in Eyes with Vitreomacular Traction and Macular Holes

**DOI:** 10.1155/2017/6834692

**Published:** 2017-01-04

**Authors:** Quraish Ghadiali, Sarwar Zahid, Rosa Dolz-Marco, Anna Tan, Michael Engelbert

**Affiliations:** ^1^Department of Ophthalmology, Manhattan Eye Ear and Throat Hospital of Northwell Health, 210 East 64th Street, New York, NY 10021, USA; ^2^Vitreous Retina Macula Consultants of New York, 460 Park Avenue, New York, NY 10022, USA; ^3^Department of Ophthalmology, New York University Langone Medical Center, 462 1st Avenue, New York, NY 10016, USA; ^4^Singapore National Eye Center and Singapore Eye Research Institute, The Academia, 20 College Road, Discovery Tower Level 6, Singapore 169856

## Abstract

*Purpose*. To compare the stages of vitreous degeneration in patients with vitreomacular traction (VMT) and macular holes (MH).* Methods*. A retrospective study was performed analyzing stages of vitreous degeneration of eyes with VMT or MH using swept-source optical coherence tomography (SS-OCT) and spectral-domain optical coherence tomography (SD-OCT). An analogous review was performed on a control group of eyes with contralateral posterior vitreous detachments. Thirty-four eyes with VMT/MH and 39 control eyes were reviewed.* Results.* Twenty-seven VMT/MH eyes and 31 control eyes were included. Eyes with VMT/MH demonstrated significantly earlier stages of vitreous degeneration when compared to the control group (*p* = 0.048) despite significantly greater age (*p* = 0.032).* Conclusions*. Vitreoretinal interface disease is more often associated with a formed vitreous and an intact premacular bursa. This is contrary to previous assumptions implicating degeneration of vitreous as a precipitating factor of interface disease when in conjunction with abnormal vitreomacular separation.

## 1. Introduction

Multimodal imaging has promoted a better understanding of vitreoretinal interface disease. Visualization of the structural alterations of the retina and the vitreoretinal interface has changed both the diagnosis and management of vitreomacular traction (VMT) and macular holes (MH). However, our understanding of the role of the vitreous body itself in this spectrum of disease has been neglected. As a result, it is typically accepted that progression of vitreous degeneration may be responsible for disease in an eye with a pathologically adherent cortical vitreous [1]. However, this theory has not been substantiated by in vivo imaging modalities.

The idea that vitreous degeneration may not, in fact, be positively correlated with vitreoretinal interface disease originated from the authors' anecdotal observations during VMT and MH repair by pars plana vitrectomy. During manipulation of the posterior vitreous, it was noted that it generally appeared to be elastic, formed, and gel-like rather than exhibiting the mobility and displacement one would expect in degenerated vitreous that may be encountered during detachment repair in patients of similar age. This was further observed on optical coherence tomography imaging leading to further investigation.

The purpose of this study was to analyze the vitreous body and its grade of degeneration in eyes with VMT and MH.

## 2. Materials and Methods

This retrospective observational case series was conducted at Vitreous Retina Macula Consultants of New York. The study complied with the Health Insurance Portability and Accountability Act and adhered to the tenets of the Declaration of Helsinki. The Western Institutional Review Board ruled that approval was not required for this retrospective study.

### 2.1. Subjects

A retrospective chart review was performed on patients who presented with VMT or MH as a manifestation of an anomalous PVD to a single physician (ME) between February 2013 and November 2015 (VMT/MH group). The diagnosis of VMT or MH was based on fundus examination and either SS-OCT or SD-OCT imaging.

A second analogous retrospective chart review was also performed to establish a control group consisting of patients presenting with an uneventful, “normal,” unilateral posterior vitreous detachment (PVD) and an attached vitreous in the contralateral eye. These contralateral eyes without a PVD were placed in the control group. Patients with pathologic myopia, lamellar holes, and neovascular disease were excluded. Furthermore, eyes with a history of vitreoretinal surgery were also excluded.

### 2.2. Data Collection

Our current understanding of posterior vitreous anatomy has recently been augmented by swept-source optical coherence tomography (SS-OCT) which allows an enhanced depth of field [2–4]. Furthermore, manipulation of image capture using standard spectral-domain optical coherence tomography (SD-OCT) allows for improved visualization of structures within the posterior vitreous [5, 6]. We previously described the anatomical features seen within the posterior vitreous and proposed a grading scheme for vitreous degeneration based on the state of the premacular bursa and its relationship to the various lacunae and degenerative cleavage planes within the vitreous body [2]. The study defined grade 0 as having an intact premacular bursa with no connections to more anterior spaces within the vitreous. Grade 1 identified anterior cleavage planes with speckled hyperreflectivity without connection to the premacular bursa. More advanced vitreous degeneration was seen in grade 2, which was characterized by connections between these spaces and the premacular bursa. Finally, grade 3 degeneration displayed connections between the premacular bursa and larger anterior lacunae.

All eyes underwent a complete ophthalmic examination as well as either structural SD-OCT or SS-OCT macular scans using the Heidelberg Spectralis (Heidelberg Engineering, Heidelberg, Germany) or the DRI OCT-1 Atlantis SS-OCT (Topcon Medical Systems, Oakland, NJ), respectively, using protocols described by our group previously [2, 7]. Briefly, radial scans of the vitreous were taken with SD-OCT using defocus, scan-tilt, and automatic real-time averaging. SS-OCT scans were completed using 5-line cross-scan patterns focused on the vitreous and contrast adjusted using the viewing software to enhance visualization of vitreous structures. Scans were excluded if the premacular bursa was not readily visible. For each eye, all B-scans through the central macula from both the VMT/MH and control groups were exported. A single nonblinded evaluator (QG) familiar with the hypothesis of the study graded each eye based on the previously described grading scheme established by our group. The exported B-scans from both study groups were then randomized and two additional blinded evaluators (RDM, AT), who were not aware of the hypothesis of the study, graded each eye.

Eyes were excluded from the data analysis if there was disagreement in grading between all three evaluators and/or scan quality did not allow unequivocal grading. If two or more evaluators were in agreement, the eye was assigned the corresponding grade and was included in the data analysis. Therefore, disagreement by only the nonblinded grader would not exclude an eye from the final analysis.

### 2.3. Statistical Analysis

Average ages between the control groups were compared using a two-tailed heteroscedastic *t*-test. The distributions of vitreous grades between the control and VMT/MH groups were compared using a Fisher exact test of a 4 by 2 contingency table. Statistical analyses were performed using SPSS v.22.0 (IBM Corp., Armonk, NY, USA). A *p* value less than 0.05 was considered statistically significant.

## 3. Results

A total of 34 eyes in the VMT/MH group and 39 eyes in the control group were reviewed for the study. After excluding eyes with disagreement between evaluators, 27 eyes were included in the VMT/MH group and 31 eyes in the control group. Average age in the VMT/MH group (68.2 years, range: 45–93) was higher than the control group (63.4, range: 50–77) (*p* = 0.0137, *t*-test). Regarding eyes with a previous history of cataract surgery, the two groups were not significantly different (VMT/MH = 5 eyes, control = 2 eyes, *p* = 0.2332, Fisher exact test).

**Figure 1:**
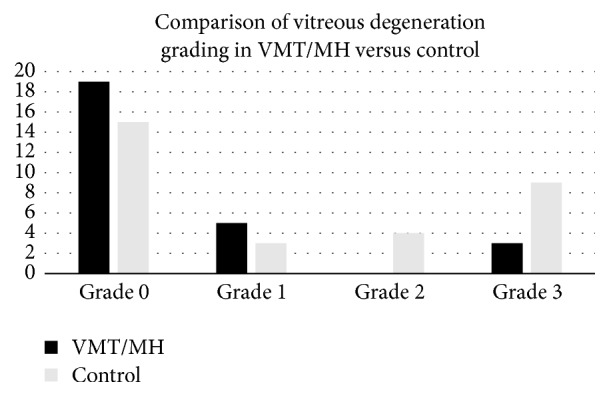
Distribution of vitreous grades in vitreomacular traction/macular hole (VMT/MH) eyes and control eyes. The VMT/MH eyes demonstrated significantly earlier degrees of vitreous degeneration when compared to the control group (*p* = 0.048).

**Figure 2:**
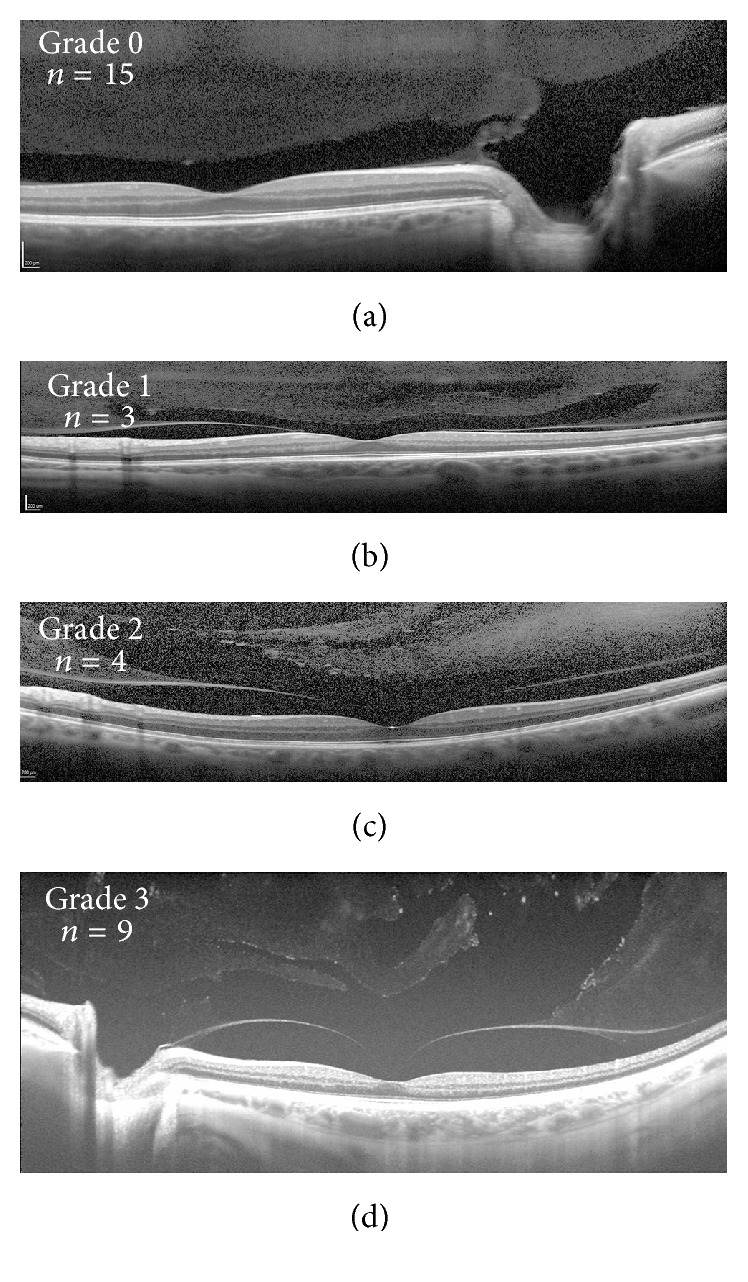
Examples of vitreous grading in eyes from patients with a posterior vitreous detachment in the contralateral eye. Spectral-domain optical coherence tomography (a to c) and swept-source optical coherence tomography (d) examples of grade 0 (a), 1 (b), 2 (c), and 3 (d) vitreous degeneration. Number of eyes demonstrating each vitreous grade included in respective panels.

**Figure 3:**
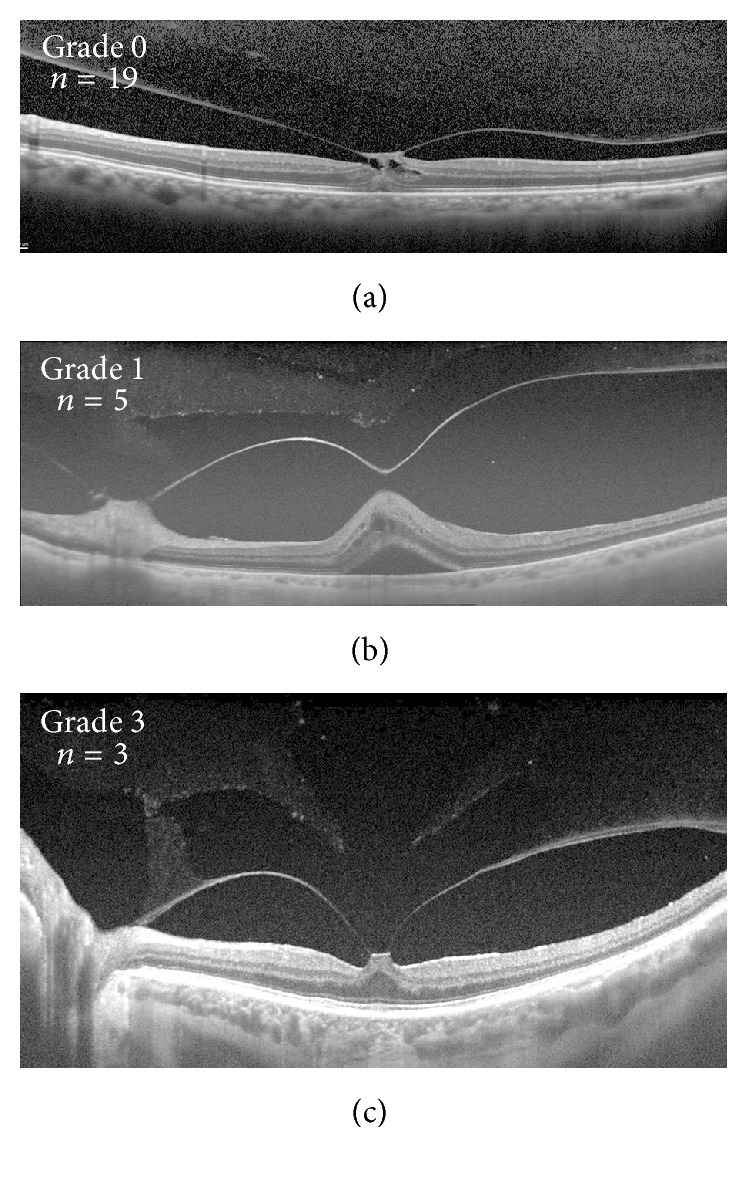
Examples of vitreous grading in eyes from patients with vitreomacular traction/macular hole (VMT/MH). Spectral-domain optical coherence tomography (a) and swept-source optical coherence tomography (b and c) examples of grade 0 (a), 1 (b), and 3 (c) vitreous degeneration. Number of eyes demonstrating each vitreous grade included in respective panels. Note that no patient in the VMT/MH group demonstrated grade 2 vitreous degeneration.

Of the 27 eyes in the VMT/MH group, 7 had MH and 20 had VMT.  Table 1 displays patient characteristics for each eye and their respective vitreous degeneration grading while Figure 1 demonstrates the distribution of vitreous degeneration grades within each group. Both groups exhibited varying degrees of degeneration with grade 0 representing the highest frequency in both groups. The levels of vitreous degeneration demonstrated in the VMT/MH group were 19 grade 0 (70.4%), 5 grade 1 (18.5%), and 3 grade 3 (11.1%). Grade 2 vitreous degeneration was not seen in this group. The levels of vitreous degeneration demonstrated in the control group were 15 grade 0 (48.4%), 3 grade 1 (9.7%), 4 grade 2 (12.9%), and 9 grade 3 (29.0%). Examples of each grade within the control and VMT/MH group are pictured in Figures 2 and 3, respectively.

A Fisher exact test showed a statistically significant difference between the VMT/MH and control groups (*p* = 0.048) demonstrating earlier stages of vitreous degeneration within the VMT/MH group.

## 4. Discussion

In this study, we assessed the stages of vitreous degeneration in patients with VMT and MH. When compared to a control group, eyes with VMT and MH demonstrated a statistically significant deviation toward earlier stages of vitreous degeneration. As the VMT/MH group was significantly older than the control group, this correlation is unlikely to be confounded by vitreous degeneration related to age. Our data reveal that grade 0 has highest prevalence in both groups but the latter stages of degeneration vary considerably.

**Figure 4:**
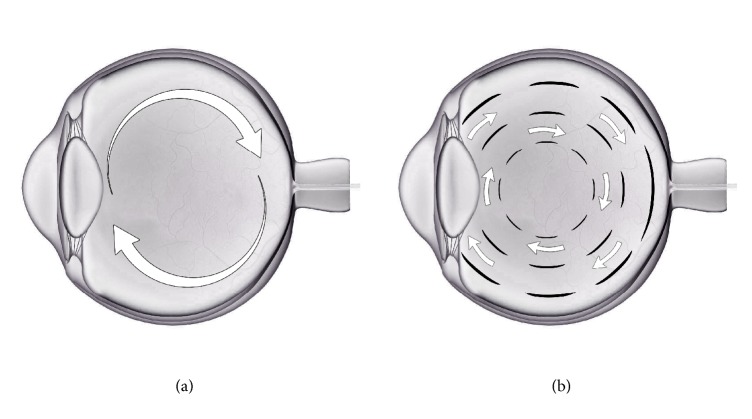
Vitreous degeneration force diagram: Shown is a schematic horizontal section of an eye with early stages of vitreous degeneration with formed vitreous (a). The forces are transmitted to the vitreoretinal interface by more central, formed vitreous without buffering from compartmentalization. Advanced vitreous degeneration with compartmentalization of the formed vitreous by liquid spaces may buffer the transmission of these forces (b).

While an abnormally strong adhesion of the posterior cortical vitreous to the macula is thought to play an important role in VMT/MH formation, the above findings suggest that a relatively formed vitreous anteriorly may also be involved in the pathogenesis of MH and VMT. Although seemingly counterintuitive and contrary to previous thinking [1], the pathogenesis involved may be substantiated by existing mechanical models. While it is known that rotation of the eye results in shearing forces on the retina transmitted from the vitreous gel, saccadic movements also cause a secondary motion of the vitreous itself directed inward to the axis of rotation. This force is in a direction opposite to the pull of the extraocular muscles exerting inertia counterforce at the vitreoretinal interface (Figure 4(a)) [8, 9]. Therefore, degeneration and compartmentalization of the vitreous gel may buffer or interrupt transmission of inertia counterforce exerted by the more central vitreous (Figure 4(b)). Spaide first suggested that the preexisting liquid spaces in the vitreous may function as a buffer, but since they are universally present, this would not explain why some eyes develop VMT or MH, while others do not [10]. As demonstrated in this study, vitreous degeneration is variable. The observation that a more formed vitreous is present in eyes affected by VMT or MH as observed during pars plana vitrectomy repair by the senior author and the OCT correlation presented in this study support the notion that vitreous degeneration or rather a lack thereof is implicated in vitreoretinal interface disorders.

Several of the limitations of this study stem from its retrospective nature and small sample size. A stratified analysis of VMT and MH did not demonstrate a statistically significant difference compared to controls, likely secondary to limited subject numbers in each respective disease group. In addition, one observer was familiar with the observations that lead to the study hypothesis and may have introduced unintentional bias. However, disagreement by the two additional blinded graders would likely negate this bias. Finally, while eyes with previous vitreous surgery were excluded, other intraocular surgeries such as cataract extraction may have disturbed the vitreous architecture leading to an altered state of the vitreous. Given that the proportion of pseudophakic patients was not significantly different between groups, this would be an unlikely confounder.

It is probable that a constellation of factors contribute to disease at the vitreoretinal interface. While the data presented show a significant correlation between interface disease and formed vitreous, three eyes demonstrated advanced vitreous degeneration. Since MH can occur when only residual cortex is present as seen in eyes after pars plana vitrectomy or in myopic macular holes with a preexisting PVD, it is clear that the forces generated to create MH and VMT are not derived exclusively from the vitreous body [11].

However, this study suggests that progression of vitreous degeneration is not a causative factor in vitreoretinal interface disease but may in fact be preventative. The implications of this theory may alter the way we think about not only the pathogenesis but also future treatment paradigms of VMT-MH spectrum disorders. It may be possible that enzymatic mechanisms used to advance vitreous degeneration may slow or halt the progression of vitreoretinal interface disease. Conceivably, the degree of vitreous degeneration is a currently overlooked prognostic factor in spontaneous resolution of VMT and MH with persistently attached vitreous, or in the success of enzymatic vitreolysis. This may be translated even further to consider prophylactic advancement of vitreous degeneration in the contralateral vitreous of a diseased eye. In the same vein, the state of vitreous degeneration may alter the rate of enzymatic vitreolysis and consequently guide treatment strategies for VMT and MH. Future prospective investigations may allow us to understand the unfolding of vitreous degeneration in patients with vitreoretinal interface disease.

## 5. Conclusions

Multiple factors are likely to contribute to the pathogenesis of vitreoretinal interface disturbance along the VMT-MH spectrum. Modern imaging methods such as SD-OCT and SS-OCT allow for enhanced visualization of vitreous evolution and will continue to broaden our understanding of retinal disease.

This study is the first to support the novel hypothesis that the state of the vitreous itself may play a role in addition to the strength of vitreoretinal adhesion.

## Figures and Tables

**Table 1a:** (a) VMT/MH group

Age	Gender	Diagnosis	Grade
73	F	VMT	0
61	M	VMT	0
68	M	VMT	0
65	F	MH	0
59	F	VMT	0
69	F	VMT	0
79	M	VMT	0
68	F	VMT	0
93	F	VMT	0
81	F	VMT	0
63	F	MH	0
78	F	MH	0
65	M	VMT	0
62	F	VMT	0
69	M	VMT	0
66	M	MH	0
74^*∗*^	F	VMT	0
68	F	MH	0
73	F	VMT	0
56	M	VMT	1
73	F	VMT	1
66	F	VMT	1
45	F	MH	1
67	F	VMT	1
75	F	VMT	3
73	F	MH	3
74^*∗*^	F	VMT	3

VMT: vitreomacular traction.

MH: macular hole.

M: male; F: female.

^*∗*^Both eyes included from a single patient.

**Table 1b:** (b) Control group

Age	Gender	Grade
68	F	0
69	F	0
61	F	0
71	F	0
57	M	0
77	M	0
70	F	0
54	F	0
62	F	0
68	M	0
51	F	0
61	F	0
73	M	0
61	M	0
64	F	0
71	F	1
67	F	1
73	M	1
63	F	2
63	F	2
68	M	2
57	F	2
65	F	3
59	M	3
75	F	3
61	M	3
50	M	3
68	F	3
58	M	3
58	M	3
60	M	3

M: male; F: female.
